# Scene-Adaptive Loader Trajectory Planning and Tracking Control

**DOI:** 10.3390/s25041135

**Published:** 2025-02-13

**Authors:** Yingnan Li, Wenwen Dong, Tianhao Zheng, Yakun Wang, Xuefei Li

**Affiliations:** 1 Key Laboratory of CNC Equipment Reliability, Ministry of Education, School of Mechanical and Aerospace Engineering, Jilin University, Changchun 130022, China; lyn_jlu@163.com (Y.L.); 13039045770@163.com (T.Z.); wyk0474@163.com (Y.W.); 2 XCMG Construction Machinery Co., Ltd., Xuzhou 221004, China; dongwenwen@xcmg.com

**Keywords:** wheel loader, dynamic grid maps, hierarchical tracking control, trajectory planning and control

## Abstract

Wheel loaders play a crucial role in daily production and transportation. With the rapid development of intelligence in passenger vehicles, freeing loader operators from high-risk and repetitive tasks has become a pressing issue. This paper presents a novel and efficient path planning and tracking framework tailored to the unique body structure and specific operating environment of loaders. We improve the Hybrid A* search algorithm based on the operational characteristics of loaders and integrate it with dynamically updated grid maps to enable the autonomous planning of loader operating paths in unstructured environments, meeting the efficiency requirements of production. Additionally, to address the challenge of poor trajectory tracking control accuracy caused by hydraulic articulated steering, we propose a new loader trajectory tracking controller based on the idea of hierarchical control. We use an extended state observer to compensate for unknown disturbances in the steering execution layer and employ fuzzy fractional-order PID to handle the nonlinearity of loaders. Field experiments using the proposed approach demonstrate that loaders can autonomously and in real-time complete tasks in dynamically changing operating scenarios.

## 1. Introduction

Wheel loaders are widely used in unstructured terrains such as mines, mixing stations, and construction sites due to their unique articulated steering structure [1], which provides greater flexibility during transportation. The unmanned autonomous operation of wheel loaders has garnered significant attention from scholars, given challenges like harsh working environments and high operational repeatability. However, the wheel loader’s unique steering structure and specialized operational scenarios render traditional passenger vehicle trajectory planning and tracking algorithms unsuitable. Thus, developing adaptive trajectory planning and tracking control solutions tailored to loader operational scenarios is crucial for achieving unmanned loader operations [2,3].

The foundation of autonomous operation lies in the rational planning of a feasible path for the loader. W. Yossawee et al. [4] proposed a semi-optimal path generation scheme comprising three line segments and two clothoid curves. This scheme demands high symmetry in the loader’s operating path, a condition challenging to maintain in the real world. Alshaer et al. [5] extended and improved the shortest path calculation method of the Reeds–Shepp algorithm to propose a V-shaped path generation scheme. However, the use of fixed-radius arcs hinders the ability to plan a reasonable path when facing boundary constraints in operating scenarios. Choi and Huhtala [6] addressed this challenge by employing Bezier curves as feasible motion primitives and combining them with an online search algorithm like A* to achieve loader trajectory generation in semi-structured environments. While this scheme enhances the loader’s adaptability to the scene, its real-time performance still falls short of meeting the efficiency requirements of loader production. Chen et al. [7] proposed a method that combines dynamic window path planning with model predictive control (MPC) to help articulated vehicles working in underground tunnels avoid collisions on their own. This strategy effectively addresses the obstacle avoidance difficulties encountered by articulated vehicles traversing limited pathways.

Despite previous research, achieving a loader motion trajectory that adapts to both the environment and operational conditions still requires addressing several challenges. Firstly, during loader operations, new obstacles may appear (such as pedestrians or other cooperative vehicles), while existing obstacles may disappear (such as diminishing material piles). Secondly, the planned path must adhere to loader kinematic constraints. This paper presents a method based on the improved Hybrid A* algorithm with dynamic grid maps. This method combines global navigation and local planning layers to allow for the real-time planning of autonomous loader operation paths in environments that are not structured.

Ensuring the safe, efficient, and accurate tracking of predefined trajectories is a critical aspect of enhancing the autonomous operation capabilities of loaders. Nayl et al. [8,9] used an improved sliding mode control and model predictive control algorithm based on the nonlinear motion model of loaders to achieve the lateral control of articulated vehicles. Bai et al. [10] proposed a nonlinear model predictive control scheme to enhance the tracking performance of articulated vehicles at high speeds, and validated the effectiveness of their approach under simulation conditions. Chen et al. [11] improved path tracking on uneven terrain by adding iterative learning as a feedforward control component to the current MPC framework. This made the MPC control more accurate. The precision of control depends on the ongoing optimization of a singular trajectory through iterative learning. Wei et al. [12] solved the problem of route tracking on surfaces with low traction by creating a hierarchical cascaded control (HCC) framework that estimates the ground adhesion coefficient. Chen et al. [13] showed a model predictive control (MPC) algorithm that uses fuzzy switching logic to help articulated vehicles handle different road conditions and speeds better. Yu et al. [14] studied how to improve the parameters for articulated wheel loader route tracking using LQR by testing how well different intelligent clustering algorithms (AGA, QPSO, and ACA) worked with LQR. This gave us a lot of information for future research. Wang et al. [15] came up with a reinforcement learning-based control strategy for loaders’ front and back axles that makes it easier to follow a path. However, this method is not very useful in real life because it does not take into account how topography and changes in load affect tracking performance.

After examining the aforementioned contributions, the subsequent observations might be articulated: (1) Rarely has the literature examined the practical performance of route tracking control techniques for articulated loaders. (2) The issues with keeping an eye on large curves and handling sudden changes in curves, which are common in the real world, have not been looked into in depth. (3) In the past, researchers have mostly ignored the influence of pressure fluctuations and disturbances in hydraulic steering systems on tracking performance. The main contributions of this paper are as follows:

(1) To address the unique scenarios of loader operations, an improved Hybrid A* planning algorithm based on dynamic grid maps is proposed, enhancing the adaptability of trajectory planning.
(2) To improve loader tracking performance, a hierarchical trajectory tracking control scheme is employed. Firstly, in the upper-layer control, a fuzzy fractional-order PID controller with preview control incorporating curvature speed adaptation is selected. Secondly, to mitigate the impact of the unique articulated steering structure on tracking, a PD controller based on a nonlinear extended state observer (NLESO) is utilized in the lower-layer control.
(3) The application of the proposed planning and control scheme to actual vehicles has been supported by a large number of experimental results, which thoroughly demonstrate the effectiveness of this strategy.

The remaining sections of this paper are organized as follows: Section 2 establishes the kinematic model of the loader and provides a detailed introduction to the characteristics of autonomous loader operations. In Section 3, an improved Hybrid A* planning scheme based on dynamic grid maps is presented. Section 4 details the trajectory tracking control scheme for the loader. Section 5 presents specific experimental results and discussions. Finally, Section 6 provides a summary of this paper.

## 2. Kinematic Modeling and Problem Statement

### 2.1. Kinematic Modeling

**Figure 1:**
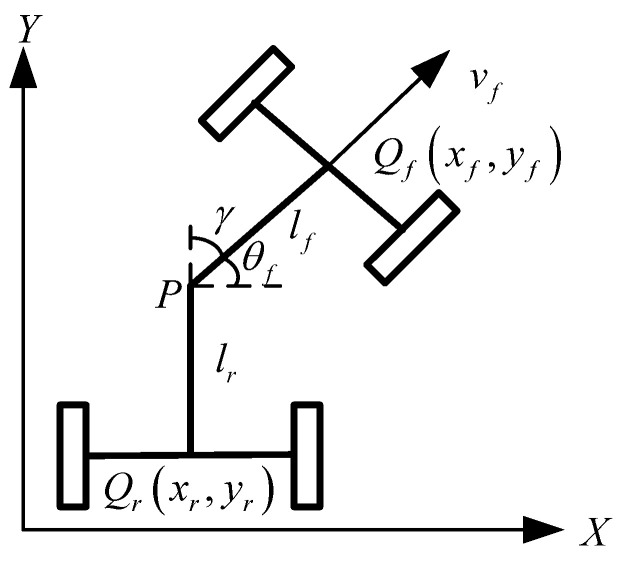
Loader kinematics model.

As shown in Figure 1, the mechanical structure of an articulated loader differs from conventional passenger vehicles. It utilizes an articulated steering device to divide the vehicle into front and rear sections, enabling more flexible steering by adjusting the angle of the joint (articulated angle γ). This design is particularly advantageous for operations in narrow terrains. The vehicle structure described in this paper is extensively detailed and analyzed in [16], which explains the kinematics of the loader. The kinematic model of the loader can be represented as

(1) $$\left[\begin{array}{c} {\dot{x}}_{f}\\ {\dot{y}}_{f}\\ {\dot{\theta }}_{f}\\ \dot{\gamma } \end{array}\right]=\left[\begin{array}{c} \cos\theta_{f}\\ \sin\theta_{f}\\ \frac{\sin\gamma }{l_{f}\cos\gamma +l_{r}}\\ 0 \end{array}\right]v_{f}+\left[\begin{array}{c} 0\\ 0\\ \frac{l_{r}}{l_{f}\cos\gamma +l_{r}}\\ 1 \end{array}\right]\omega_{r}$$

### 2.2. Problem Statement

**Figure 2:**
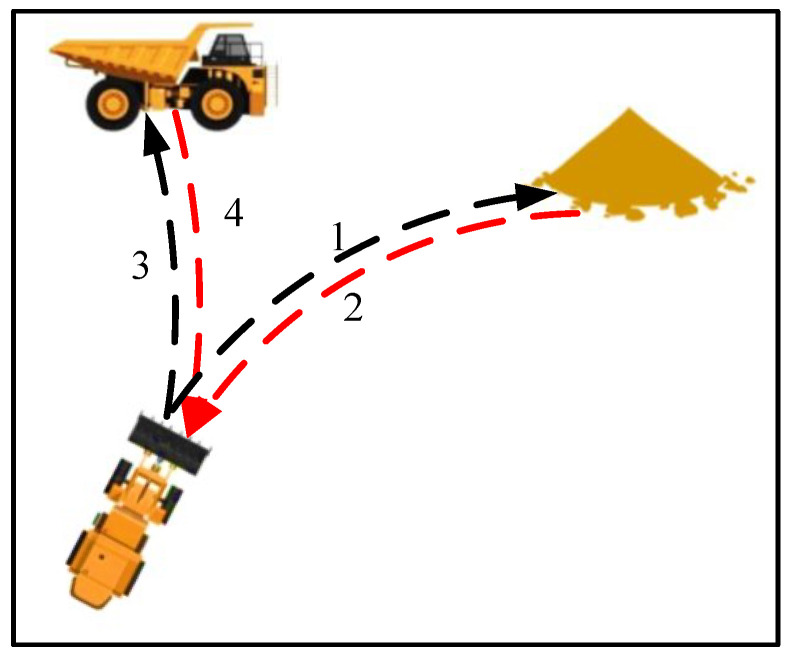
Transportation conditions of loaders.

When operating in transportation tasks, V-shaped trajectories are favored by many drivers due to the characteristic of short operating cycles and high efficiency [17]. The specific workflow of this operating condition is illustrated in Figure 2. Firstly, the loader advances to the loading point and loads materials (trajectory 1); then, it reverses to the turning point (trajectory 2); next, it moves forward to the unloading point (such as a truck or hopper) and unloads materials (trajectory 3); and finally, it reverses back to the turning point (trajectory 4).

## 3. Trajectory Planner

During actual loader operations, changes in obstacle information can render paths planned based on global navigation maps inadequate for safe passage. While some scholars have addressed similar issues using quadratic programming algorithms [18], the specialized nature of loader operating scenarios, such as the need for long-term parking of cooperative vehicles like self-dumping trucks, requires the re-execution of quadratic programming after each global planning, impacting loader operational efficiency. Additionally, traditional dynamic search algorithms [19], which require significant trial and error time in the initial planning stage, are unsuitable for the point-to-point operation mode of loaders.

**Figure 3:**
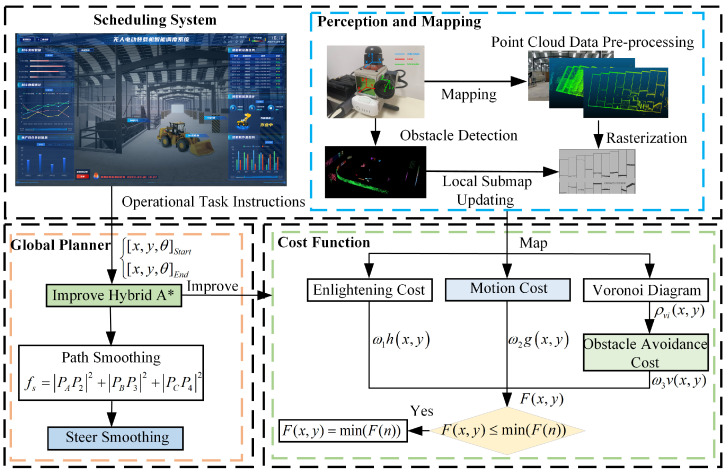
Loader trajectory planning process.

To address these challenges, this paper first plans the loader’s global navigation path using an improved Hybrid A* algorithm based on a pre-established global navigation map. During loader operation, real-time updates of local grid maps are performed. These updates, combined with the global navigation path, allow for the identification of obstacle information affecting loader passage and determining the need for re-planning. The updated grid map is then applied in the next global navigation planning cycle. The specific planning process is illustrated in Figure 3.

### 3.1. Path Planning Based on Improved Hybrid A* Algorithm

To address the phenomenon that the A* algorithm [20] performs poorly when dealing with planning problems in the presence of motion constraints, Dolgov et al. [21] proposed a hybrid A* search algorithm with heuristic components to solve incomplete constraints.

#### 3.1.1. Collision Avoidance

In the global planning process, it is crucial to consider not only the length of the path but also its effectiveness in obstacle avoidance. To achieve this, distance maps are commonly employed [22]. However, when dealing with narrow passages in loader operating scenarios, this approach may lead to sub-optimal solutions and fail to plan a viable path.

In recent years, Voronoi diagrams [23] have been widely applied in obstacle avoidance path planning. While this method does not directly yield the shortest path between two points, it partitions obstacles into cells, allowing for the maximum utilization of gaps between obstacles to generate a drivable path that stays as far away from obstacles as possible. To ensure the safety and efficiency of loader operations, this paper incorporates Voronoi diagrams into the expansion of nodes in the Hybrid A* algorithm, using the resulting potential values as part of the cost function for the heuristic function. Based on the size parameters of the loader equipment, the following Voronoi potential field function is constructed:

(2) $$\rho_{v}\left(x,y\right)=\left\{\begin{array}{c} \left(\frac{\alpha }{\alpha +d_{0}\left(x,y\right)}\right)\left(\frac{d_{v}\left(x,y\right)}{d_{v}\left(x,y\right)+d_{0}\left(x,y\right)}\right){\left(\frac{d_{0}\left(x,y\right)-d_{0}^{\max}}{d_{0}^{\max}}\right)}^{2},d_{0}\leq d_{0}^{\max}\\ 0,d_{0}>d_{0}^{\max} \end{array}\right.$$

where, setting the rate of potential energy α to be decreased to 0.5, the maximum control range of potential energy $d_{0}^{\max}$ is 5 m.

#### 3.1.2. Cost Function

With the above potential energy values as safety terms, the cost function is designed as follows:

(3) $$F\left(x,y\right)=w_{1}g\left(x,y\right)+w_{2}h\left(x,y\right)+w_{3}v\left(x,y\right)$$

where $w_{1}$, $w_{2}$, and $w_{3}$ are the weighting factors, $g\left(x,y\right)$ indicates the cost of movement from the initial point to the current point, $h\left(x,y\right)$ represents the estimated cost from the current point to the termination point, $v\left(x,y\right)$ is the cost of avoiding obstacles, and $F\left(x,y\right)$ is the total cost value.

Referring to the trajectories of experienced drivers, when planning the motion trajectory of a loader, it is important to ensure that the loader moves forward as much as possible to avoid frequent steering changes that could lead to path fluctuations, while also minimizing the number of turning maneuvers. Therefore, this paper designs the motion cost function as follows:

(4) $$g\left(x,y\right)=P_{fs}l_{fs}+P_{ft}l_{ft}+P_{bs}l_{bs}+P_{br}l_{br}+P_{rev}l_{rev}$$

where $l_{fs}$ is the total step length for forward straight movement, $l_{ft}$ is the total step length for forward turning, $l_{bs}$ is the total step length for backward straight movement, $l_{bt}$ is the total step length for backward turning, and $l_{rev}$ is the number of turning maneuvers. $P_{fs}$, $P_{ft}$, $P_{bs}$, $P_{bt}$ and $P_{rev}$ are the corresponding cost coefficients for each form.

The heuristic cost $h\left(x,y\right)$ is defined as follows:

(5) $$h\left(x,y\right)=\max\left(\begin{array}{c} \mathrm{Cos}t_{WithoutObst}\\ \mathrm{Cos}t_{WithObst} \end{array}\right)$$

Here, $\mathrm{Cos}t_{WithoutObst}$ ignores the influence of obstacles in the environment, connects the current point to the target point through a Reeds–Shepp curve, and calculates the length of the optimal curve as the heuristic cost value, but $\mathrm{Cos}t_{WithObst}$ is necessary to consider the information of obstacles in the environment, while also taking into account the influence of the motion direction of parent and child nodes, and determining the new cost value by executing the Hybrid A* algorithm.

The loader exhibits a ‘bending’ phenomenon during actual movement, and a detailed description can be found in this reference [24]. Therefore, to ensure that the loader does not experience collision accidents during operation, the obstacle avoidance cost $v\left(x,y\right)$ should be designed as follows:

(6) $$\begin{array}{c} v\left(x,y\right)=\max\left(\rho_{v1}\left(x,y\right),\rho_{v2}\left(x,y\right),\rho_{v3}\left(x,y\right),\rho_{v4}\left(x,y\right)\right.,\\ \left.\rho_{v5}\left(x,y\right),\rho_{v6}\left(x,y\right),\rho_{v7}\left(x,y\right),\rho_{v8}\left(x,y\right)\right) \end{array}$$

When the vehicle reaches point $\left(x,y\right)$, $\rho_{vi}\left(x,y\right)$ represents the Voronoi potential values of the eight corners in the bounding boxes of the front and rear bodies of the loader. To ensure safe driving, the obstacle avoidance cost should be set as the maximum potential value among them.

### 3.2. Path Post-Processing

#### 3.2.1. Smoothing Process

The path generated by the planning program consists of numerous discrete points, forming an initial planned path that may not ensure smooth vehicle travel along the designated route in the operating environment. Therefore, this paper employs gradient descent to smooth the initial path. The specific optimization principles and process are detailed below.

**Figure 4:**
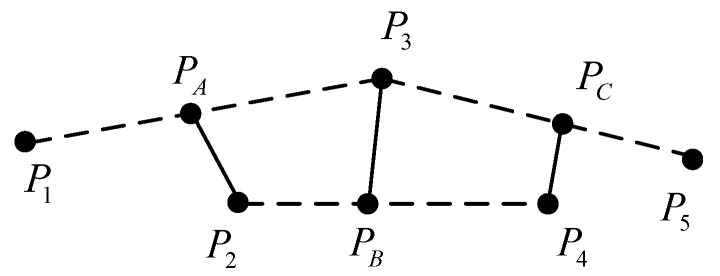
Path smoothing principle.

Smoothness is defined as shown in Figure 4, where $P_{1}∼P_{5}$ is the five discrete points in the initial planning path, $P_{A}$ is the midpoint of $P_{1}$ and $P_{3}$, $P_{B}$ is the midpoint of $P_{2}$ and $P_{4}$, and $P_{C}$ is the midpoint of $P_{3}$ and $P_{5}$. Therefore, the smoothing degree is calculated as

(7) $$f_{s}={\left|P_{A}P_{2}\right|}^{2}+{\left|P_{B}P_{3}\right|}^{2}+{\left|P_{C}P_{4}\right|}^{2}$$

From Figure 4, it is evident that the smoothness changes with the position $P_{3}$, and there is always a position $P_{3}$ that minimizes the smoothness. Therefore, the optimized position points can be obtained using the gradient descent algorithm. Initially, the optimal displacement ΔP of $P_{3}$ is set in the gradient direction. By setting the first derivative of smoothness with respect to ΔP to zero, ΔP can be calculated using the following formula:

(8) $$\begin{array}{cc} \frac{\partial f_{s}}{\partial \Delta P} & =\frac{{\left(P_{1}+P_{3}+\Delta P-2P_{2}\right)}^{2}+{\left(P_{2}+P_{4}-2P_{3}-2\Delta P\right)}^{2}}{\partial \Delta P}\\ & +\frac{{\left(P_{3}+\Delta P+P_{5}-2P_{4}\right)}^{2}}{\partial \Delta P} \end{array}$$

(9) $$\Delta P=-\frac{1}{6}\left(P_{1}-4P_{2}+6P_{3}-4P_{4}+P_{5}\right)$$

Moving the path points according to (9) and iterating through multiple calculations, a fully smooth path can be obtained.

#### 3.2.2. Path Connection at Turning Points

Analysis of the optimized smooth driving trajectory reveals that at turning points, the loader’s articulated steering, which connects the front and rear bodies, can cause an abrupt change in the heading of the front body, leading to unstable vehicle control [5]. In practical tracking tests of articulated vehicles, this instability often manifests as serpentine movements and poor tracking accuracy during subsequent tracking, significantly limiting the loader’s tracking performance. To address these issues, this paper introduces a straight line segment at the turning point, equal in length to the distance between the front and rear axles of the vehicle. The specific implementation is as follows.

Extract the coordinates and heading angle at the turning point $\left(x_{rev},y_{rev},\theta_{rev}\right)$ based on the turning flag. Assuming the length of the straight line segment added at this point is *L* and it contains *N* path points, the distance between adjacent path points is $\frac{L}{n}$. Therefore, the coordinates of the i_th path point can be calculated as follows:

(10) $$\left\{\begin{array}{c} x\left(i\right)=x_{rev}-i\cdot \frac{L}{n}\cdot \cos\theta_{rev}\\ y\left(i\right)=y_{rev}-i\cdot \frac{L}{n}\cdot \sin\theta_{rev}\left(1\leq i\leq n\right)\\ \theta \left(i\right)=\theta_{rev} \end{array}\right.$$

Substituting i=1∼n into (10) provides the coordinates for all path points along the connecting straight line path. These points are then sequentially inserted after the turning point $\left(x_{rev},y_{rev},\theta_{rev}\right)$ to complete the path connection at the turning point.

## 4. Trajectory Tracking and Control

**Figure 5:**
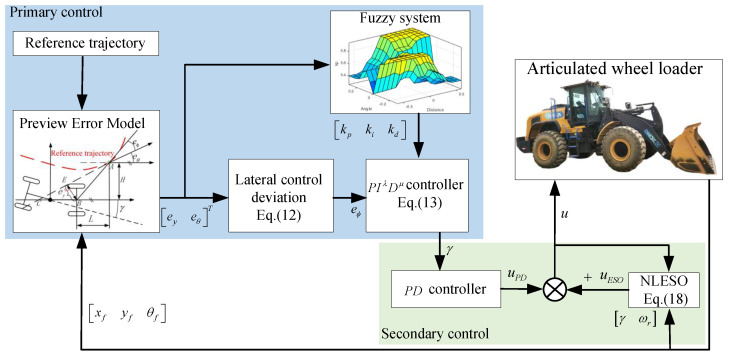
Loader trajectory tracking control framework.

To ensure the loader can follow the planned trajectory, this paper proposes a hierarchical controller for its path tracking. Figure 5 illustrates the system framework. Initially, the trajectory tracking layer designs a preview error model with curvature and speed adaptive capabilities. This model enhances tracking precision using a fuzzy fractional-order PID controller. Next, this study uses a PD controller based on the nonlinear extended state observer (NLESO) to solve the problem of the steering actuator not being able to accurately respond to the upper control layer’s steering demands when disturbances are unknown.

### 4.1. Preview Error Model

**Figure 6:**
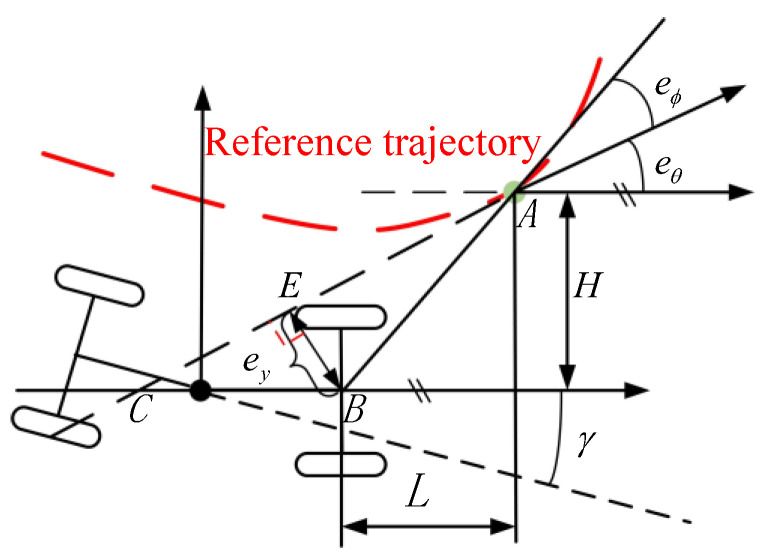
Loader preview error model.

By analyzing the driving habits of loader operators, it is evident that drivers typically concentrate their attention on an imagined “target point” in front of the vehicle, known as the “look-ahead point” [25]. Referring to driver behavior, when there is no driver in the loader, it is also necessary to provide a look-ahead point to help the controller better track the planned trajectory. The specific articulated vehicle preview error model is shown in Figure 6.

The selection of the conventional preview distance [26] is typically based solely on changes in the vehicle speed, which results in poor adaptability to changes in curvature. Considering that loaders often track large curvature and significant curvature changes at turning points in “V” shaped operating curves, ensuring the stability and smoothness of loader travel, this paper introduces a curvature factor into the original preview distance formula to adjust the impact of curvature on the tracking accuracy. The specific curvature and speed-adaptive preview distance adjustment algorithm is shown in (11):

(11) $$L=\left\{\begin{array}{ll} L_{\min} & v<v_{\min}\\ L_{0}+\frac{k_{1}v}{k_{2}S+\sigma } & v_{\min}\leq v\leq v_{\max}\\ L_{\max} & v>v_{\max} \end{array}\right.$$

Considering the safety of the loader, when the speed exceeds the specified maximum speed $v_{\max}$, it is usually kept in a straight line, so only the maximum preview distance $L_{\max}$ when the curvature is 0 needs to be considered. At the same time, through a large number of experiments, it has been proven that when the speed is lower than the minimum travel speed $v_{\min}$, the curvature has little effect on the performance of the preview control [27] and is typically based solely on changes in the vehicle speed, which results in poor outcomes. Therefore, only the minimum preview distance $L_{\min}$ should be set according to the conventional preview distance formula. When the speed is in the range of $v_{\min}\leq v\leq v_{\max}$, this paper introduces the weight factor $k_{2}$ and σ to reflect the degree of correlation between the degree of road curvature and the preview distance, and the weight factor $k_{1}$ is used to reflect the degree of correlation between the speed and the preview distance. The parameters of the preview error model are set as follows: $v_{\min}=0.5$, $v_{\max}=2$, $L_{\min}=1.74$, $L_{0}=1.2$, $k_{1}=1.08$, $k_{2}=40$, σ=1, and $L_{\max}=3.36$.

### 4.2. Lateral Control Deviation

From Section 4.1, it is clear that the objective of lateral control for the unmanned loader is to ensure that the loader satisfies the conditions of $e_{y}\to 0$ and $e_{\theta }\to 0$. Here, the preview lateral deviation $e_{\phi }$ is defined as shown in Figure 6. Based on this, Theorem 1 can be formulated.

**Theorem** **1** *The entire proof is divided into the following two parts.*

*Part 1: Proving that* $e_{\phi }\to 0\Rightarrow e_{y}\to 0,e_{\theta }\to 0$ *.*

*In Figure 6,* $e_{\phi }=∠BAC$ *(Lateral control deviation). Meanwhile, in* ΔABC *,* $\sin e_{\phi }=\frac{BE}{AB}$ *. Since* $e_{\phi }\to 0$ *and* $AB\geq L\geq d_{\min}\neq 0$ *, it can be inferred that* BE→0 *, i.e.,* $e_{y}\to 0$ *. Moreover, because* $\sin∠ACB=\frac{BE}{BC}$ *and* BE→0 *, it can be inferred that* $∠ACB=e_{\theta }\to 0$ *. Consequently, the conclusion is proven.*

*Part 2: Proving that* $e_{\phi }\to 0⇐e_{y}\to 0,e_{\theta }\to 0$ *. Since in* ΔABC *,* $\sin e_{\phi }=\frac{BE}{AB}$ *, if* BE→0 *, it has* $e_{\phi }\to 0$ *. The conclusion is proven.*

In summary, lateral control deviations are defined as follows:

(12) $$e_{\phi }=\arctan\left(\frac{H\left(e_{y},e_{\theta }\right)}{L}\right)-e_{\theta }$$

### 4.3. Fuzzy Fractional-Order PID Controller

Loaders typically operate on unstructured surfaces, with the uncertainty of the objects being handled and the unique hydraulic articulated steering. These factors make loaders highly nonlinear, uncertain, and subject to time delays. In certain highly nonlinear and uncertain systems, conventional PID controllers may fail to deliver satisfactory tracking performance, particularly when precise path tracking is essential. Therefore, this paper proposes improvements to the PID controller by introducing fractional-order PID and fuzzy control.

#### 4.3.1. Fractional-Order PID Controller

The transfer function of the FOPID controller is expressed as follows [28]:

(13) $$C_{\left(s\right)}=k_{p}+\frac{k_{i}}{s^{\lambda }}+k_{d}s^{\mu }$$

Here, $k_{p}$, $k_{i}$ and $k_{d}$ are the corresponding gain coefficients, all of which are positive. To ensure effective control execution, it is necessary to perform a finite-order approximation of the FO-differ-integral within a specific frequency range. This paper adopts Outstaloup’s fractional calculus finite approximation technique [29]. Specifically, when the frequency range is $\left(\omega_{b},\omega_{h}\right)$, the filter’s transfer function is expressed as follows:

(14) $$G_{filter}\left(s\right)=\omega_{h}^{\beta }\overset{n}{\underset{l=-n}{\Pi }}\frac{s+{\omega^{\prime }}_{l}}{s+\omega_{l}}$$

(15) ωl′=ωb(ωhωhωbωb)l+N+ll−β22N+1

(16) ωl=ωb(ωhωhωbωb)l+N+ll+β22N+1

where 2N+1 and β represent the orders of the filter and the integrator, respectively.

#### 4.3.2. Fuzzy Rules

To simplify the design process, this paper optimizes the fractional-order parameters λ and μ based on experience. As a result, the fuzzy control system for the wheel loader only has two input variables and three output variables. The input variables are the deviations in preview distance $e_{y}$ and preview heading angle $e_{\theta }$, while the output variables are the three gain parameters of the FOPID2D controller.

**Figure 7:**
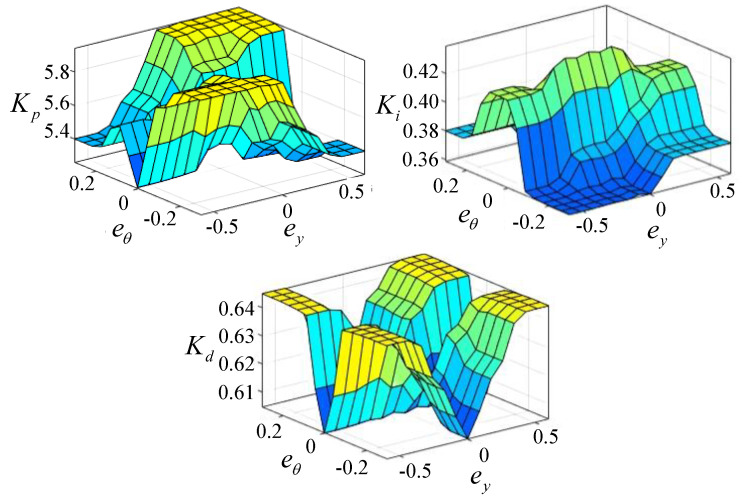
Relationship between inputs and outputs of a fuzzy controller.

It is very important to come up with the right fuzzy intervals so that the loader can quickly adjust to the right position and orientation and then fine-tune within a small range to find the best balance between the response speed and control accuracy. Through preliminary experimental tests, the corresponding optimization interval ranges are shown in Table 1. We divide fuzzy sets into five levels: NB (Negative Big), NS (Negative Small), Z (Zero), PS (Positive Small), and PB (Positive Big). Consequently, the fuzzy rules for the construction of the control system utilizing linguistic variables are presented in Table 2. Figure 7 illustrates the relationship between the input and output of the loader’s path tracking fuzzy controller.

### 4.4. Extended State Observer

Following the hydraulic steering system [30], the system state space can be described as

(17) $$\left\{\begin{array}{c} {\dot{x}}_{1}=x_{2}\\ {\dot{x}}_{2}=d\left(x_{1},x_{2},t\right)+bu\\ y=x_{1} \end{array}\right.$$

where $\left[\begin{array}{cc} x_{1} & x_{2} \end{array}\right]=\left[\begin{array}{cc} \gamma & \omega_{r} \end{array}\right]$, $\delta \left(x_{1},x_{2},t\right)$ denotes the unknown perturbation. The expression for estimating the unknown perturbation in nonlinear ESO is as follows:

(18) $$\left\{\begin{array}{c} e_{1}={\hat{x}}_{1}-x_{ref}\\ {\dot{\hat{x}}}_{1}={\hat{x}}_{2}-\beta_{1}e\\ {\dot{\hat{x}}}_{2}={\hat{x}}_{3}-\beta_{2}fal\left(e,\alpha ,\delta \right)+bu\\ {\dot{\hat{x}}}_{3}=-\beta_{3}fal\left(e,\alpha ,\delta \right) \end{array}\right.$$

In the ESO, $x_{3}=d\left(x_{1},x_{2},t\right)$ represents the extended state variable used to estimate the total sum of unknown disturbances and model errors. To mitigate its impact on control performance, $u_{ESO}=b\cdot {\overset{⌢}{\;x}}_{3}$ is introduced into the original control law:

(19) $$\begin{array}{c} fal\left(e,\alpha ,\delta \right)=\left\{\begin{array}{cc} \frac{e}{\delta^{1-\alpha }} & \left|e\right|\leq \delta \\ {\left|e\right|}^{\alpha }sign\left(e\right) & \left|e\right|>\delta \end{array}\right. \end{array}$$

where $\beta_{1}=3\omega_{0}$, $\beta_{1}=3\omega_{0}^{2}$, $\beta_{1}=\omega_{0}^{3}$, *α*, *ω*_0_, and *δ* are parameters of ESO.

## 5. Results and Discussion

**Figure 8:**
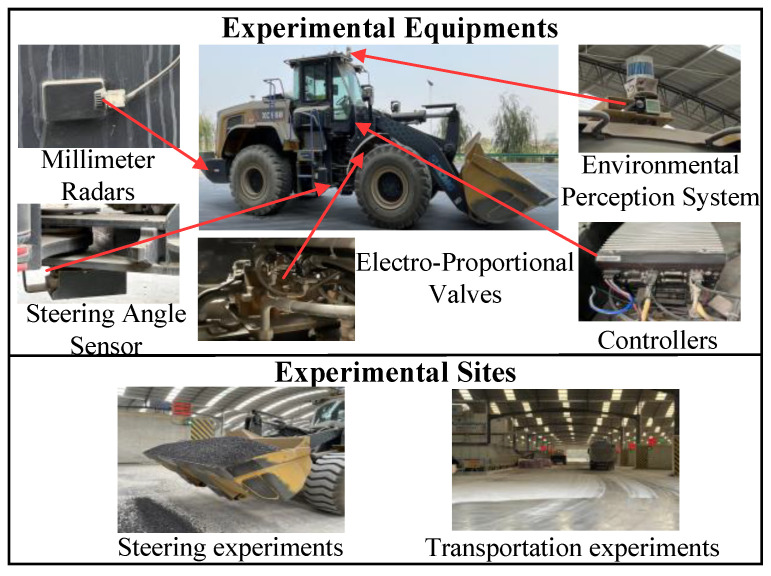
Experimental equipment and sites.

We conducted experiments using a 5 ton loader in a mixing plant scenario to validate the effectiveness of the proposed trajectory planning and tracking control strategy. The experimental setup and platform are depicted in Figure 8. This section aims to address three key questions: (1) Can the trajectory planner generate collision-free and dynamically feasible trajectories in complex operational environments in real-time (see Section 5.1)? (2) Can the hydraulic steering controller withstand unknown disturbances and meet the steering requirements of the upper control layer (see Section 5.2)? (3) How does the trajectory tracking controller perform in real-world scenarios (see Section 5.3)? The parameters of the proposed controller are presented in Table 3.

### 5.1. Planning Performance

**Figure 9:**
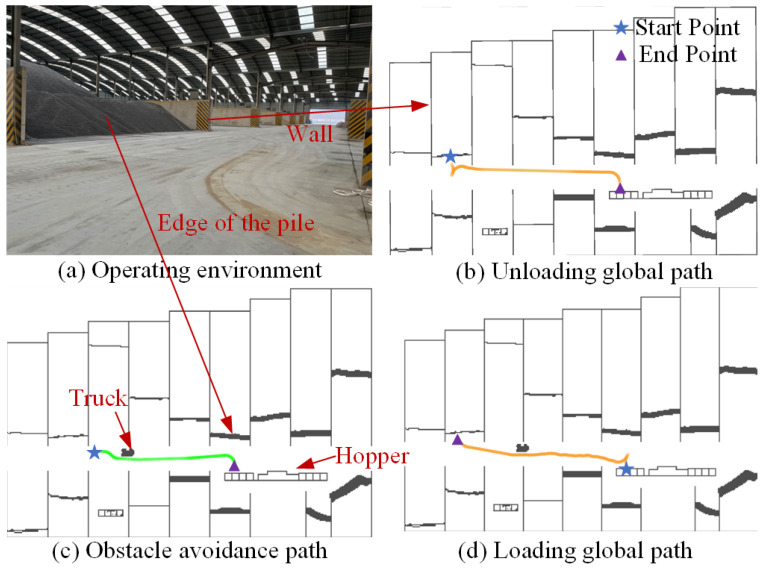
Loader loading–unloading trajectory.

This paper evaluates the proposed planning module in a real transportation scenario shown in Figure 9a. The loader’s LiDAR captures the obstacle point cloud, which we project onto the ground to create the black lines in Figure 9b–d. First, Figure 9b shows the loader planning a drivable unloading path based on the previously established global navigation map before transportation. Secondly, Figure 9c demonstrates that during driving, the loader encounters a temporary parking obstacle (a truck) and can effectively plan an avoidance path. Finally, in Figure 9d, the loader generates a reasonable shovel avoidance path based on the updated grid map.

### 5.2. Steering Performance

To address the second question, this paper evaluates the performance of the proposed controllers using two steering trajectories. Considering that loaders are primarily affected by front-end loads during steering, we examine the basic performance of the controllers under varying loads.

**Figure 10:**
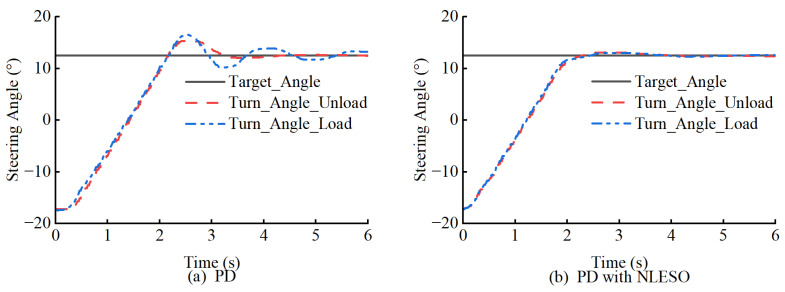
Step steering condition.

**Figure 11:**
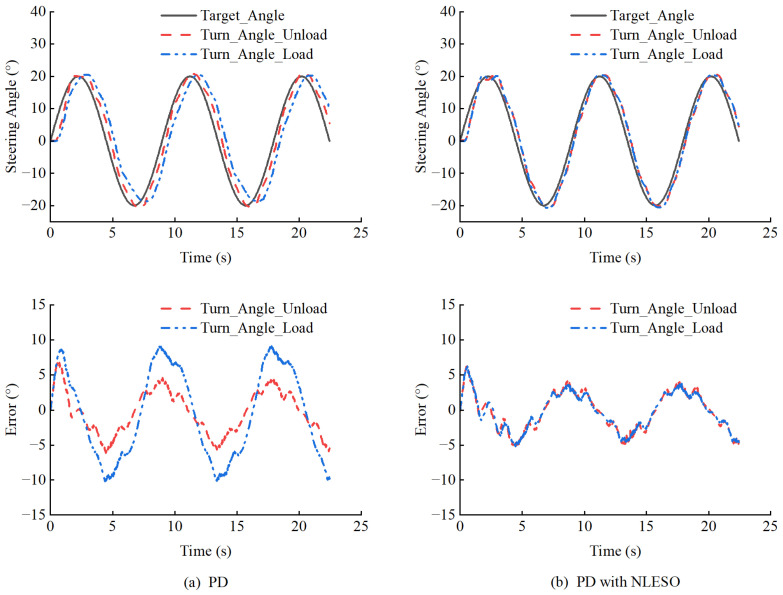
Sinusoidal steering condition.

The results indicate that the PD controller with NLESO performs better. Overall, introducing NLESO can effectively compensate for the effects of unknown disturbances on steering. As shown in Figure 10 and Figure 11, even under full load conditions, the controller with NLESO can maintain performance similar to that without additional loading. Conversely, without disturbance compensation, the tracking error significantly increases. Table 4 evaluates the performance of steering tracking sinusoidal curves using ME, MAE, and RMSE.

### 5.3. Trajectory Tracking Performance

This study evaluates the proposed framework by demonstrating the controller’s superiority and efficacy through tests and simulations.

#### 5.3.1. Simulation Results

To validate the efficacy of the proposed path tracking system in curvature challenges, a dual-shift path is formulated as follows:

(20) $$\left\{\begin{array}{c} Y_{ref}\left(x\right)=\frac{d_{n1}}{2}\left(1+\tanh\left(2\cdot r_{1}\right)\right)-\frac{d_{n2}}{2}\left(1+\tanh\left(2\cdot r_{2}\right)\right)\\ \theta_{ref}\left(x\right)=\arctan\left(d_{n1}{\left(\frac{1}{\cosh\left(r_{1}\right)}\right)}^{2}\left(\frac{1.2}{d_{m1}}\right)-d_{n2}{\left(\frac{1}{\cosh\left(r_{2}\right)}\right)}^{2}\left(\frac{1.2}{d_{m2}}\right)\right) \end{array}\right.$$

where $d_{m1}=35$, $d_{m2}=40$, $d_{n1}=7$, $d_{n2}=9$, $r_{1}=\frac{4}{d_{m1}}\left(x-27.19\right)-1.2$ and $r_{2}=\frac{4}{d_{m2}}\left(x-54.46\right)-1.2$.

**Figure 12:**
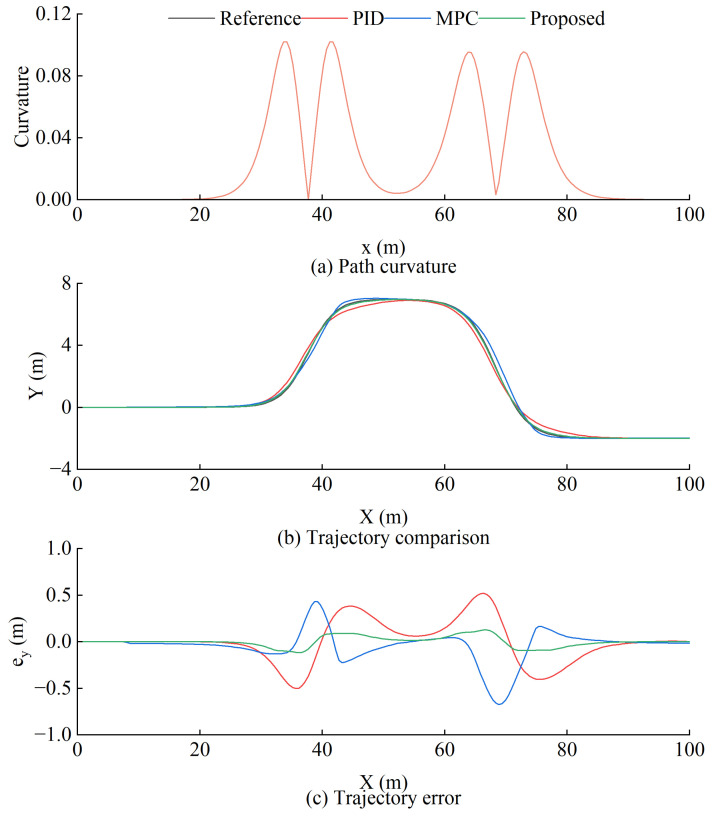
Simulation results for the dual-shift path.

**Figure 13:**
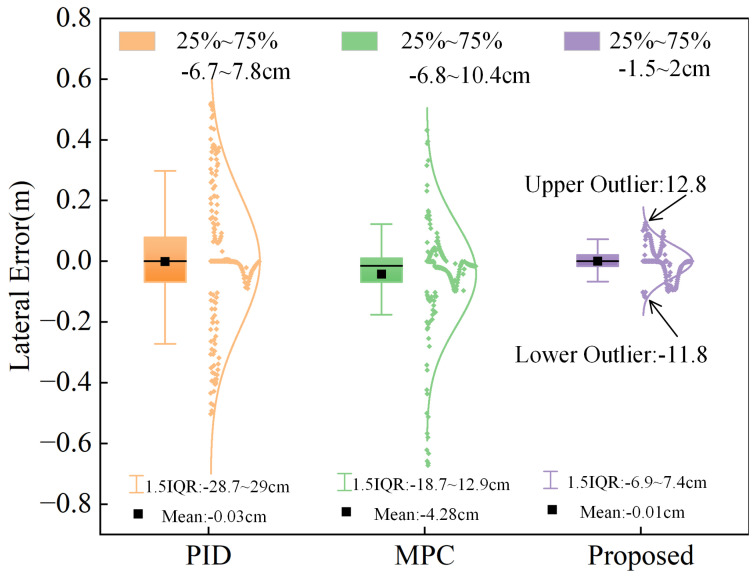
Box plot of simulation results.

Figure 12a illustrates that the path curvature is variable over time, reaching a maximum of 0.1. When comparing with popular path tracking controllers (PID and MPC), it is clear that while MPC has rolling prediction capabilities that allow it to maintain good tracking performance during scopes with slow changes in curvature, its maximum error may rise to 67 cm in scopes with sudden changes in curvature. This fails to comply with the safety standards for loader operation. Although the proposed strategy in this work leads to a bigger error within the curvature mutation interval, the biggest error stays at just 12.8 cm, which is a lot less than the other two controllers. To evaluate the steady-state performance of the controller, Figure 13 illustrates that the error of the proposed control scheme mainly lies within the range of −1.5 to 2 cm, while the interquartile range (IQR) is from −6.9 to 7.4 cm, which means that the error is more concentrated. The outlier range is limited to −11.8 to 12.8, thereby ensuring the loader’s operational safety.

#### 5.3.2. Experimental Results

As shown in Figure 8, this experiment aimed to demonstrate that the proposed planning approach can generate drivable paths in real-time and achieve tracking in challenging operational conditions. The system can resist the effects of unknown disturbances and maintain good tracking performance.

**Figure 14:**
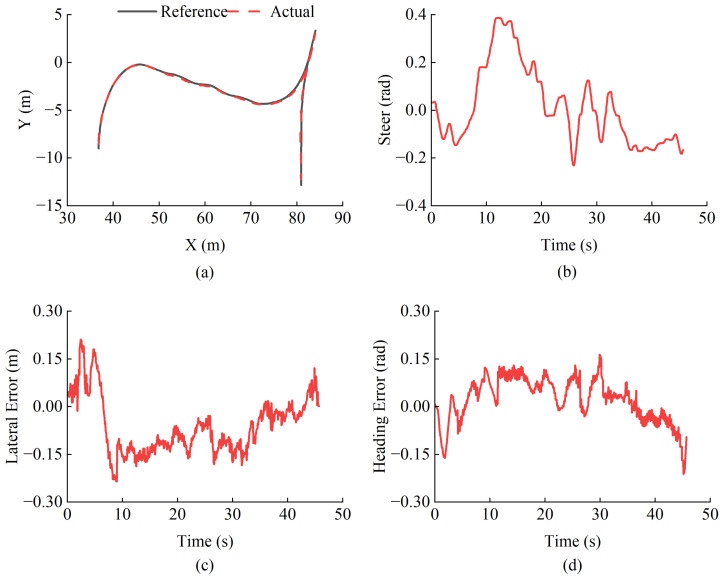
Trajectory tracking results (**a**) Trajectory comparison (**b**) Control input (**c**) Lateral error (**d**) Heading error.

The tracking performance in the real world is shown in Figure 14, where the position error is generally within ±0.15 m, with a maximum error of −0.235 m, and the heading error ranges from −0.21 rad to 0.16 rad. As shown in Figure 14b, the articulation angle remains stable throughout the tracking process, with an output range within ±0.4 rad, which is less than the loader’s steering constraint angle.

## 6. Conclusions

This paper presents a practical solution for planning and tracking in an unmanned loader system. By dynamically updating the grid map and using an improved Hybrid A* algorithm, the system achieves obstacle avoidance and transportation trajectory planning in loader operation scenarios. We also propose a layered controller with disturbance compensation to precisely track predefined motion trajectories. Extensive experiments have confirmed the effectiveness of this strategy. Based on the proposed strategy, our future research will focus on addressing the steering actuator delay and enhancing the adaptability of the trajectory tracking control scheme. Furthermore, we plan to further explore new control strategies and investigate how data-driven approaches can be integrated with traditional control techniques to address more complex environments and tasks.

## Figures and Tables

**Table 1:** Fuzzy control optimization interval ranges.

Output Parameters	Range	Input Parameters	Range
$k_{p}$	[5.20, 6.00]	$e_{y}$	[−0.6, 0.6]
$k_{i}$	[0.36, 0.42]	$e_{\theta }$	[−0.3, 0.3]
$k_{d}$	[0.60, 0.65]		

**Table 2:** Fuzzy rules.

$k_{p}/k_{i}/k_{d}$		$e_{y}$				
		NB	NS	Z	PS	PB
$e_{\theta }$	NB	$k_{p5}/k_{i1}/k_{d5}$	$k_{p4}/k_{i1}/k_{d4}$	$k_{p4}/k_{i2}/k_{d2}$	$k_{p3}/k_{i2}/k_{d3}$	$k_{p2}/k_{i1}/k_{d4}$
	NS	$k_{p4}/k_{i1}/k_{d3}$	$k_{p3}/k_{i2}/k_{d3}$	$k_{p3}/k_{i3}/k_{d2}$	$k_{p2}/k_{i2}/k_{d3}$	$k_{p1}/k_{i1}/k_{d4}$
	Z	$k_{p3}/k_{i3}/k_{d2}$	$k_{p2}/k_{i4}/k_{d1}$	$k_{p1}/k_{i5}/k_{d1}$	$k_{p2}/k_{i4}/k_{d1}$	$k_{p3}/k_{i3}/k_{d2}$
	PS	$k_{p1}/k_{i1}/k_{d3}$	$k_{p4}/k_{i2}/k_{d3}$	$k_{p3}/k_{i3}/k_{d2}$	$k_{p3}/k_{i2}/k_{d3}$	$k_{p4}/k_{i1}/k_{d3}$
	PB	$k_{p2}/k_{i1}/k_{d4}$	$k_{p3}/k_{i1}/k_{d4}$	$k_{p4}/k_{i2}/k_{d2}$	$k_{p4}/k_{i1}/k_{d4}$	$k_{p5}/k_{i1}/k_{d5}$

**Table 3:** Controller parameters.

Module	Parameter Settings
Path planning	$w_{1}=1,w_{2}=1,w_{3}=10,p_{fs}=1,p_{ft}=1.3,p_{bs}=2,p_{br}=2,p_{rev}=5$
Path tracking	$\lambda =0.86,\mu =1.32,\alpha =0.5,\omega_{0}=5.2,\delta =0.05,b=14.5$

**Table 4:** Steering performance under sinusoidal conditions.

		ME	MAE	RMSE
	PD	7.11	2.67	0.29
Unload	PD with NLESO	6.24	2.37	0.22
	PD	10.2	5.64	0.43
Load	PD with NLESO	6.26	2.22	0.22

## Data Availability

Data will be made available on request.
